# Editorial: Risk-Based Evidence for Animal Health Policy

**DOI:** 10.3389/fvets.2020.00595

**Published:** 2020-09-04

**Authors:** Lisa A. Boden, Harriet K. Auty, Amy Delgado, John D. Grewar, Amy D. Hagerman, Thibaud Porphyre, George C. Russell

**Affiliations:** ^1^The Global Academy of Agriculture and Food Security, The Royal (Dick) School of Veterinary Studies, The Roslin Institute, University of Edinburgh, Midlothian, United Kingdom; ^2^Boyd Orr Centre for Population and Ecosystem Health, Institute of Biodiversity, Animal Health and Comparative Medicine, College of Medical, Veterinary and Life Sciences, University of Glasgow, Glasgow, United Kingdom; ^3^United States Department of Agriculture, Animal Plant and Health Inspection Service, Veterinary Services, Strategy and Policy, Center for Epidemiology and Animal Health, Fort Collins, CO, United States; ^4^South African Equine Health and Protocols NPC, Cape Town, South Africa; ^5^Department of Agricultural Economics, Oklahoma State University, Stillwater, OK, United States; ^6^The Royal (Dick) School of Veterinary Studies, The Roslin Institute, University of Edinburgh, Midlothian, United Kingdom; ^7^Moredun Research Institute, Pentlands Science Park, Midlothian, United Kingdom

**Keywords:** risk, evidence, science-policy, zoonoses, COVID-19, risk assessment, animal health, epidemiology

Infectious animal and zoonotic diseases are important and immediate global disease threats which exhaust resources and place demands on both national and international global animal and human health institutions and infrastructures. These diseases create challenges for industry stakeholders and policy-makers because of their pandemic potential and resultant widespread economic and social disruption. The current pandemic of the coronavirus SARS-CoV-2 (disease name COVID-19), which was first detected in the wet markets of Wuhan, Hubei Province, China, offers a contemporary example on which we might reflect about the lessons learned from this Research Topic—in particular, the importance of transparent data sharing and the development of risk-based evidence for policy-making for zoonotic disease outbreak preparedness and control. COVID-19 has now been detected in 188 international locations despite the closure of the wet markets and imposition of movement restrictions and other interventions to reduce risks of onward transmission. Risk management decisions in different countries [such as the imposition and subsequent release of social distance policies (1) and the introduction of compulsory mask-wearing (2)] are not purely (public health) science-based. The political, cultural, and societal dimensions of the pandemic have highlighted sharply the need to “remedy… disciplinary silos” (3) through holistic interdisciplinary approaches to understand the complex trade-offs and unintended consequences of disease control policies.

In this Research Topic, we wanted to explore the development of a robust and fit-for-purpose evidence base for animal (and public) health and the different mechanisms used to ensure its effective delivery to policy-makers in order to better anticipate and respond appropriately to existing and emerging animal and zoonotic disease risks. The response to the call for papers yielded 17 accepted papers with 112 contributing authors and the Research Topic has been accessed more than 25,000 times highlighting the importance and timely nature of these contributions. In this editorial, we identify 5 key lessons learned from these contributions and consider the future for risk-based policy-making for animal and public health.

## Improve Understanding and Communication of Concepts of Risk and Uncertainty To Different Stakeholder Audiences

Policy and decision-making is not based on scientific evidence alone, but also influenced by political will, existing governance structures, public opinion, and other exogenous factors. Researchers need to engage with all of these facets in a holistic way, but this is challenging to do in the context of traditional research environments. More reflects on these difficulties and highlights the need for a commitment to integrate “policy relevance to the research focus from the outset, to engage with policy-makers and other stakeholders throughout, to use platforms to facilitate science-policy dialogue, and to disseminate research findings appropriately.” He articulates the need and demand for interdisciplinary approaches—and in particular, input from the social sciences, which stems from the recognition that science, itself, is not value-free.

Assembling multi-disciplinary teams with appropriate expertise is fundamental to delivering appropriate and effective risk assessment, communication, and management. Countries have varying approaches to prioritizing disease risks for contingency planning which reflect the economic, social, and cultural values of their communities. Two papers in this series explore risk prioritization and perception, through different disciplinary approaches. Bessell et al. use a semi-quantitative approach which uses a combination of the rate of disease spread, disease mitigation factors, impacts on animal welfare and production, the human health risks and the impacts on wider society to characterize exotic disease priorities for Scotland. In contrast, Waldman et al., explore the role of the social, economic, and cultural context in shaping the perceptions and practices of actors who play significant roles in risk management. This paper illustrates the importance of understanding “situated expertise” and particular forms of risk perception and practice which both enhance and compromise risk reduction in different ways” (Waldman et al.).

## Anticipate Regulatory or Policy Barriers To Ensure Effective Implementation of Scientific Evidence

The foundations of evidence-based decision-making begin with robust data collection, access and sharing. Houe et al. acknowledge that although there may be a wealth of data generated, many datasets have emerged from different organizations and have been developed for other purposes, making it difficult to integrate them and use them to their full potential. Appropriate regulations and policies need to be in place for data access and sharing across organizational and legal boundaries. Sustaining the value of these datasets to researchers and decision-makers depends almost entirely on data accuracy and reliability; substantial changes in data architecture and structure, which inevitably occur over time, need to be taken into account to ensure that risk management decisions based on these data are justified and valid. Continued investment into the maintenance and “upkeep” of these data is therefore critical for these data to be useful to policy-makers.

## Integrate Different Data Sources To Improve Disease Monitoring and Surveillance

Estimating the risk of incursion of disease depends on transparent data sharing, robust animal health recording systems and fit-for-purpose veterinary public health infrastructure which includes access to affordable diagnostic tests, laboratory facilities, and trained technicians, veterinary professional, paraprofessionals, and researchers to interpret and act on results. Georgaki et al. describe the advantages of the Bluetongue surveillance programme in Northern Ireland, which has evolved to include the use of risk assessments and simulation models to monitor the risk of incursion. Its design enables effective mitigation measures to be identified to minimize disease risk and provides additional assurances to protect NI's export markets in the European Union (EU) and third countries. The authors also highlight the benefits of including both active and targeted surveillance activities to enable early detection of disease. In Scotland, risk-based approaches are also used to identify high risk areas for vector-borne diseases, such as Louping ill virus (See Gilbert et al.). GIS-based data on environmental variables, when used in combination with sero-prevalence data, become a powerful tool to identify risk factors and improve opportunities for identification of alternative disease reservoirs. Both of these are important for informing disease management policies and identifying trade-offs between environmental and farming priorities and costs. These insights are echoed in the contributions by Carneiro et al. and Semango et al. which remind us of the value of traditional field-based epidemiology and recognize the importance of a systems-based approach. As highlighted by the example of COVID-2019, a broad and holistic understanding of the causal risk pathways is necessary to ensure that critical disease reservoirs are also appropriately incorporated into strategies for surveillance and risk mitigation.

## Invest in Proactive Development of Risk Assessment Expertise and Generic, Flexible Tools, and Frameworks Which are Ready-To-Use in Disease Emergencies

The majority of the contributions in this Research Topic identified the usefulness of investing in proactive veterinary risk assessments which include risk pathways that can be flexibly adapted and re-used in times of emergency to ensure business continuity (see Auty et al.; de Vos et al.; Taylor et al.; Umber et al.; Walz, Middleton et al.; Walz, Evanson et al.). For example, estimates of the risk of onward transmission of disease associated with movements of carcasses from de-populated farms to other areas for disposal inform risk management decisions about movements of vehicles, animals and animal products out of disease control areas (Umber et al.; Walz, Evanson et al.). While there is a lot of guidance available for animal-related product movements and for a variety of carcass types, there may be country, disease or species-specific gaps which are necessary to fill in order to “assist regulatory authorities in using risk” to guide decision-making (for example to grant permitted movement or deny a request to move for live animals or carcasses) (see Umber et al.). Proactively working to elucidate these risks coupled with efforts to identify and address data and research gaps can help countries minimize the risk of disease spread while also minimizing the impact of the outbreak response on unaffected farms.

## Incorporate Social Science and Humanities Expertise To Improve and Discriminate Between Different Risk Management Decisions

Risk assessment is essential to target critical control points which are amenable to risk reduction. Although technical solutions—such as improved diagnostic testing regimens and disease control strategies—are available, their effectiveness depends on the compliance and uptake of interventions by key stakeholders. Two papers explored the likelihood of uptake of technological interventions, using distinct approaches. Mohr et al., used economic game theory as a framework to evaluate farmers' strategic decision-making in different contexts. The work explores the uptake of an effective diagnostic test for sheep-scab—a disease which costs more than £8 million per year to the UK industry. In theory, the benefits of control should outweigh the costs of the test. However, this paper illustrates that the likelihood of uptake depends very much on the farmer's perception of risk to the herd and whether they take a long- or short-term view of profitability. Liu et al. explore this problem in a different way. The authors construct different behavioral typologies of farmers which they refer to as: “non-adopters,” “current adopters,” or “future adopters” with respect to different technologies. Their paper suggests that in order to be successful, we need to better understand our stakeholder populations so that policies, regulatory incentives, and complementary training can be appropriately targeted to ensure effective uptake and positive behavioral change.

## A Forward Look: Create New Pathways To Improve Decision-Making

New technologies and methodologies in human and veterinary medicine, epidemiology, agricultural production systems, and business tools and approaches have the capacity to deliver large volumes of high-quality data and complex analyses to improve animal and zoonotic disease surveillance and outbreak preparedness. However, scientific evidence is usually only a small part of the evidence base for decision-makers. Incorporating these advances into policy-making can be challenging, given the silos that exist between human and animal health institutions, differences between research, policy and industry timescales, and the need to consider multiple evidence bases and different stakeholder groups. Without established effective and explicit communication channels between scientists, policy, and industry audiences, researchers will struggle to respond to policy needs with relevant research to inform decision-making in a timely and robust manner. Models of science-policy delivery through innovative, multi-disciplinary partnerships between academia, industry, and government (such as the Scottish Government Centers of Expertise, described by Boden et al.), may offer a solution, particularly when combined with purposeful communication and innovation aimed at these five lessons.

## Author Contributions

LB was responsible for the concept and writing of this manuscript. All authors listed have made a substantial, direct and intellectual contribution to the work, and approved it for publication.

## Conflict of Interest

The authors declare that the research was conducted in the absence of any commercial or financial relationships that could be construed as a potential conflict of interest.
